# CFD Simulation and Structural Optimization Analysis of Micromixing Processes in T-Shaped Microreactors

**DOI:** 10.3390/mi17020234

**Published:** 2026-02-11

**Authors:** Yongzhi Ning, Bo Wang, Runci Wang, Taihong Yan

**Affiliations:** China Institute of Atomic Energy, Beijing 102413, China; ningyongzhi@cnncmail.cn (Y.N.); wangbo01@ciae.ac.cn (B.W.); wangrunci@ciae.ac.cn (R.W.)

**Keywords:** microreactor, micromixing, computational fluid dynamics (CFD), process intensification, venturi structure

## Abstract

Microreactors offer remarkable advantages in intensifying mixing/mass transfer and hold promising prospects for industrial applications. In this study, T-shaped microreactors (TMRs) integrated with baffle, orifice-plate, and venturi structures (featuring different contraction angles) were designed. Based on the Villermaux–Dushman reaction system, three-dimensional computational fluid dynamics (CFD) models were established to simulate the fluid flow and mixing-reaction processes in these microreactors. The results demonstrate that peaks in velocity, turbulent kinetic energy, and turbulent dissipation rate consistently emerge in the confluence region of the two fluid streams. In the operating range of this study, the baffle configuration exhibits the highest micromixing performance but also induces the largest pressure drop, followed by the orifice-plate structure. Notably, the venturi structure not only enhances micromixing efficiency but also results in a minimal increase in pressure drop and eliminates flow dead zones. Specifically, the venturi structure with a 45° contraction angle achieves a balance between energy consumption and micromixing efficiency. Using the agglomeration model, the micromixing times of the microreactors with various structures were determined to range from 0.025 to 0.234 ms.

## 1. Introduction

Microreactors possess distinct advantages, including a large specific surface area, small volume, low mass transfer resistance, and facile controllability, which facilitate rapid fluid mixing/mass transfer, improved reaction efficiency, and enhanced product quality [1,2]. As a result, they have been extensively applied in various fields such as nanoparticle synthesis [3,4], organic synthesis [5,6], biomedicine [7,8], and energy generation [9,10].

Microreactors are primarily categorized into two types: active and passive. Active microreactors rely mainly on external energy inputs, such as electric fields [11], magnetic fields [12], and acoustic fields [13], to drive fluid mixing [14,15]. Tang et al. [16] and Qian et al. [17] employed electric and magnetic fields for micromixer actuation, respectively, enhancing mixing intensity by inducing eddy currents to promote convective diffusion. With the deepening of research, active microreactors are evolving toward precise regulation. Yang et al. [18] proposed a mixing strategy based on suspended magnetic microrobots, and numerical simulations verified that this approach can significantly improve mixing performance. However, active systems generally require complex external driving devices, which restricts their large-scale application. Although Kazemi et al. [19] achieved precise control of mixing quality by optimizing parameters of a periodically potential-driven electroosmotic micromixer via numerical simulations, this limitation has not been fundamentally addressed.

In contrast, passive microreactors enhance mixing through internal structural design, utilizing mechanisms such as fluid chaotic convection [20] and shear effects [21,22]. Due to their advantages of simple structure, high stability, easy integration, and low energy consumption, passive microreactors have become a research focus. Furthermore, the advancement of additive manufacturing technology provides an efficient solution for fabricating their complex structures [23]. Studies on traditional structures (e.g., Tesla structures, wall-grooved structures) are relatively mature. Both Lai et al. [24] and Yang et al. [25] adopted Tesla structures to intensify mixing in microreactors. Wu et al. [26] incorporated grooves and protrusions on the microreactor wall to disrupt the stable boundary layer flow, thereby enhancing mixing efficiency.

In recent years, novel passive structures (e.g., fractal channels, periodic disturbance units, and split-recombine structures) have emerged. Related research heavily relies on CFD simulations for mechanism analysis and structural optimization. Amrendra et al. [27] compared the mixing performance of microchannels with staggered and opposed obstacle units. Movahed et al. [28] established a prediction model for liposome properties in a periodically disturbed mixer based on response surface methodology. Shao et al. [29] investigated the mixing process of a split–recombine microreactor via numerical simulations, providing a basis for structural optimization.

Focusing on TMRs, experimental and numerical simulation studies on their micromixing performance have progressively advanced. Researchers commonly employ the Villermaux–Dushman competitive reaction system to characterize micromixing efficiency, investigating the effects of parameters (e.g., flow rate, fluid viscosity, and inlet structure) on mixing performance [30]. Abiev et al. [31] confirmed that a two-stage strong swirling microreactor exhibits superior mixing efficiency and pressure drop characteristics compared to a TMR, indicating substantial room for improving the mixing performance of TMRs. With the rapid development of computing power and simulation models, CFD has been widely used to simulate flow, mixing/mass transfer, and reaction processes, revealing underlying mechanisms and structural design principles [32,33]. Soleymani et al. [34] demonstrated through CFD simulations that structural dimensions and operating parameters significantly influence the flow and mixing performance of TMRs. Aoki et al. [35] numerically studied the effects of internal structures (e.g., channel confluence and bending) on mixing performance. Ouyang et al. [36] established a CFD model for mixing-reaction processes in a double-tube microreactor, employing the probability density function (PDF) model and finite-rate/eddy dissipation model to describe the micromixing process, respectively. This model enabled the acquisition of reactant/product distribution characteristics and micromixing efficiency. Dong et al. [37] controllably synthesized mesoporous FePO_4_nanoparticles using an ultrasound-enhanced turbulent T-junction microreactor (UTISR). Combined with experimental validation and CFD simulations, they systematically explored the synergistic effect of impinging flow and ultrasonic radiation on mesoporous structure formation. Mariotti et al. [38] confirmed that flow unsteadiness in TMRs significantly affects reaction yield. Unsteady asymmetric flow patterns can increase yield by over 30%, whereas unsteady symmetric flow patterns exert the opposite effect, offering valuable insights for process intensification.

Despite the extensive use of CFD simulations in microreactor design, most existing models inadequately address the trade-off between micromixing efficiency and energy consumption. In this study, based on the Villermaux–Dushman reaction system, three-dimensional CFD models were established to characterize the fluid flow and mixing-reaction processes in TMRs with different internal structures. The developed three-dimensional CFD models were validated using experimental data, and the model parameters were determined accordingly. Under different Reynolds number conditions, the effects of internal structures (including baffles, orifice plates, and venturi tubes with various contraction angles) on the velocity distribution, turbulent kinetic energy, turbulent kinetic energy dissipation rate, and component concentration distribution inside the microreactors were systematically investigated. Meanwhile, the pressure drop and segregation index of various microreactors were obtained. Subsequently, the characteristic micromixing time was calculated by adopting the agglomeration model.

## 2. Materials and Methods

### 2.1. Geometric Model

**Figure 1:**
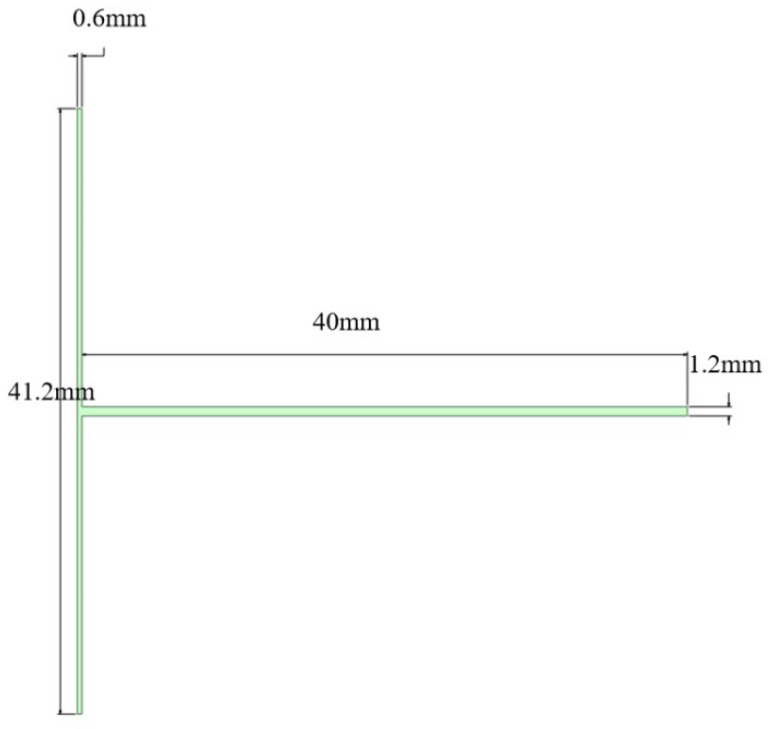
Schematic diagram of the TMR.

**Figure 2:**
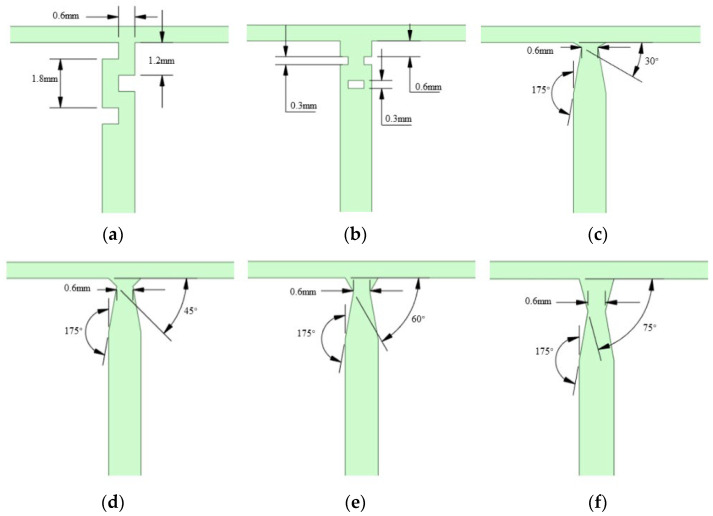
Enhanced structures of the TMR: (**a**) B-TMR; (**b**) O-TMR; (**c**) V30-TMR; (**d**) V45-TMR; (**e**) V60-TMR; (**f**) V75-TMR.

The TMR features a rectangular channel cross-section. The two inlet channels are 20 mm in length and 0.6 mm in width, while the mixing channel is 40 mm in length and 1.2 mm in width. All channels have a uniform depth of 0.6 mm (Figure 1). To enhance the mixing of the two fluid streams, baffle-type obstacles, orifice-plate structures, and venturi structures (with contraction angles of 30°, 45°, 60°, and 75°) were integrated into the mixing region. The contraction port size of all venturi structures was uniformly set to 0.6 mm. The enhanced structures and their dimensions are illustrated in Figure 2. Hereafter, the microreactors with different structures are denoted as follows: B-TMR (baffle-type), O-TMR (orifice-plate-type), V30-TMR (venturi with 30° contraction angle), V45-TMR (venturi with 45° contraction angle), V60-TMR (venturi with 60° contraction angle), and V75-TMR (venturi with 75° contraction angle).

### 2.2. Model Equations

#### 2.2.1. Governing Equations

The CFD simulation involves liquid flow and chemical reactions, requiring the solution of the continuity equation, momentum equation, and species transport equation. The reactor operates under steady-state, single-phase continuous flow conditions, and the fluid is assumed to be incompressible. The relevant governing equations are as follows:

Continuity equation:

(1) $$\nabla \cdot \left(\rho U\right)=0$$

Momentum conservation equation:

(2) $$\nabla \cdot \left(\rho UU\right)-\nabla \cdot \left[\mu \left(\nabla U+\nabla U^{T}-\frac{2}{3}\nabla \cdot UI\right)\right]=-\nabla p$$

Species transport equation:

(3) $$\nabla \cdot \left(\rho U\varphi_{\alpha }\right)=-\nabla \cdot J_{\alpha }+S_{\alpha }$$

For turbulent flow, the diffusion flux $J_{\alpha }$ is expressed as:

(4) $$J_{\alpha }=-\rho \left(D_{\alpha ,m}+\frac{\mu_{t}}{Sc_{t}}\right)\nabla \varphi_{\alpha }$$

where $\varphi_{\alpha }$ is the local mass fraction of species α; $J_{\alpha }$ is the diffusion flux of species α; $D_{\alpha ,m}$ is the mass diffusion coefficient of species α in the mixture; $Sc_{t}$ is the turbulent Schmidt number; and $\mu_{t}$ is the turbulent viscosity.

The standard *k-ε* two-equation turbulence model with an extensible wall function was used for model closure:

Turbulent kinetic energy (k) equation:

(5) $$\frac{\partial }{\partial t}\left(\rho k\right)+\frac{\partial }{\partial x_{i}}\left(\rho ku_{i}\right)=\frac{\partial }{\partial x_{j}}\left[\left(\mu +\frac{\mu_{t}}{\sigma_{k}}\right)\frac{\partial k}{\partial x_{j}}\right]+G_{k}+G_{b}-\rho \varepsilon -Y_{M}+S_{k}$$

Turbulent dissipation rate (ε) equation:

(6) $$\frac{\partial }{\partial t}\left(\rho \varepsilon \right)+\frac{\partial }{\partial x_{i}}\left(\rho \varepsilon u_{i}\right)=\frac{\partial }{\partial x_{j}}\left[\left(\mu +\frac{\mu_{t}}{\sigma_{\varepsilon }}\right)\frac{\partial \varepsilon }{\partial x_{j}}\right]+C_{1\varepsilon }\frac{\varepsilon }{k}\left(G_{k}+C_{3\varepsilon }G_{b}\right)-C_{2\varepsilon }\rho \frac{\varepsilon^{2}}{k}+S_{\varepsilon }$$

where ρ is the fluid density; $u_{i}$ is the velocity component in the $x_{i}$ direction; $G_{k}$ and $G_{b}$ are the turbulent kinetic energy generation terms due to the mean velocity gradient and buoyancy, respectively; $Y_{M}$ is the turbulent kinetic energy dissipation term caused by fluctuating expansion in compressible turbulence; $S_{k}$ and $S_{\varepsilon }$ are the source terms for k and ε, respectively. The model constants were set as follows: $C_{1\varepsilon }=1.44$, $C_{2\varepsilon }=1.92$, $\sigma_{k}=1.0$, $\sigma_{\varepsilon }=1.3$.

#### 2.2.2. Finite-Rate/Eddy Dissipation Model

For fast reaction systems, the actual reaction rate is controlled by component mixing rather than intrinsic kinetics. In the Eddy Dissipation Model (EDM), the reaction rate is determined by the smaller value between the mixing-controlled rate (calculated via EDM) and the kinetics-controlled rate (calculated via intrinsic kinetics). The species with the lowest concentration dominates the actual reaction rate.

EDM-based reaction rate:

(7) $$R_{i,r}=v_{i,r}^{\prime }M_{i}A_{m}\rho \frac{\varepsilon }{k}min\left(\frac{Y_{1}}{v_{1,r}^{\prime }M_{1}},\frac{Y_{2}}{v_{2,r}^{\prime }M_{2}},\ldots ,\frac{Y_{n}}{v_{n,r}^{\prime }M_{n}}\right)$$

Kinetics-based reaction rate:

(8) $$R_{i,r}=k_{f,r}\prod_{j=1}^{N}\;{\left(C_{j,r}\right)}^{m}-k_{b,r}\prod_{j=1}^{N}\;{\left(C_{j,r}\right)}^{n}$$

where $\nu \prime_{i,r}$ is the stoichiometric coefficient of species i in reaction r; $M_{i}$ is the molar mass of species i; $Y_{i}$ is the mass fraction of species i; $A_{m}$ is an empirical constant obtained by fitting simulation results; $k_{f,r}$ and $k_{b,r}$ are the forward and backward reaction rate constants for reaction r, respectively; and $C_{j,r}$ is the concentration of species j in reaction r.

### 2.3. Micromixing Reaction System

The Villermaux–Dushman parallel competitive reaction system [39] has been widely used for micromixing research [40,41] because of its flexibility and convenience. Based on this method, the segregation index can be defined to characterize the micromixing efficiency, analyze the micromixing processes, and optimize the operational parameters in reactors [42,43]. The iodate–iodide parallel competitive reaction system is widely used to evaluate liquid micromixing efficiency in reactors. This system includes a fast neutralization reaction, a redox reaction, and an iodine complexation reaction, with the following reaction equations:

R1 (Neutralization reaction):

(9) $$H_{2}B{O_{3}}^{-}+H^{+}\overset{k_{1}}{\to }H_{3}BO_{3}$$

R2 (Redox reaction between iodide and iodate ions):

(10) $$5I^{-}+I{O_{3}}^{-}+6H^{+}\overset{k_{2}}{\to }3I_{2}+3H_{2}O$$

R3 (Iodine complexation reaction):

(11) $$I^{-}{+I}_{2}\overset{k_{3}}{\underset{k_{4}}{⇌}}I_{3}^{-}$$

The reaction constants for R1 and R3 are $k_{1}=10^{11}\;m^{3}/(kmol\cdot s)$, $k_{3}=5.9\times 10^{9}\;m^{3}/(kmol\cdot s)$, and $k_{4}=7.5\times 10^{9}\;s^{-1}$.

At room temperature, the intrinsic kinetics of the competitive reaction (R2) between iodide and iodate ions remains controversial [44,45], but most studies classify it as a 5th-order reaction. The reaction rate is expressed as:

(12) $$r_{2}=k_{2}\left[{IO}_{3}^{-}\right]{\left[I^{-}\right]}^{2}{\left[H^{+}\right]}^{2}$$

where $k_{2}$ is the reaction rate constant, with different expressions depending on the ionic strength (I) [46]:

For ${I<0.166M,log}_{10}\left(k_{2}\right)=8.383-1.5112\sqrt{I}+0.23689$.

For $I\geq 0.166M,{log}_{10}\left(k_{2}\right)=9.28105-3.664\sqrt{I}$.

The segregation index $X_{S}$, calculated from the by-product concentration, quantifies micromixing performance:

$X_{S}=0$: Ideal mixing (only the main reaction occurs).

$X_{S}=1$: Complete segregation (only the side reaction occurs).

The calculation formulas for $X_{S}$ are:

(13) $$X_{S}=\frac{Y}{Y_{ST}}$$

(14) $$Y_{ST}=\frac{6{\left[{IO}_{3}^{-}\right]}_{0}}{6{\left[{IO}_{3}^{-}\right]}_{0}+{\left[H_{2}{BO}_{3}^{-}\right]}_{0}}$$

(15) $$Y=2\frac{\left(V_{\mathrm{buffer}\;}+V_{\mathrm{acid}\;}\right)\left(\left[I_{2}\right]+\left[I_{3}^{-}\right]\right)}{V_{\mathrm{acid}\;}\left({\left[H^{+}\right]}_{0}\right)}$$

In the experiment, the main reactant *H*_2_*BO*_3_^−^ is in excess. If mixing is complete, *H*^+^ is fully consumed by the main reaction, and no *I*_2_ or *I*_3_^−^ is generated ($X_{S}$ = 0). Otherwise, both R2 and R3 will occur, and the *X_S_* ranges between 0 and 1.

### 2.4. Mesh Model and Mesh Independence Verification

**Figure 3:**
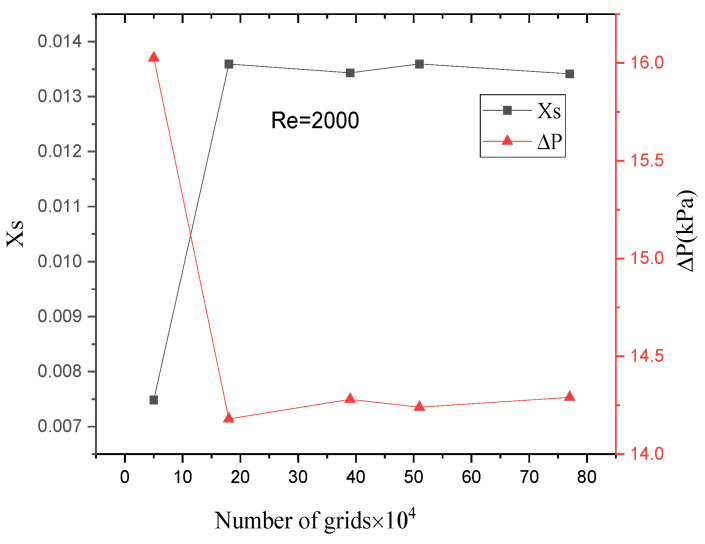
Mesh independence verification of the TMR at *Re* = 1800.

**Figure 4:**
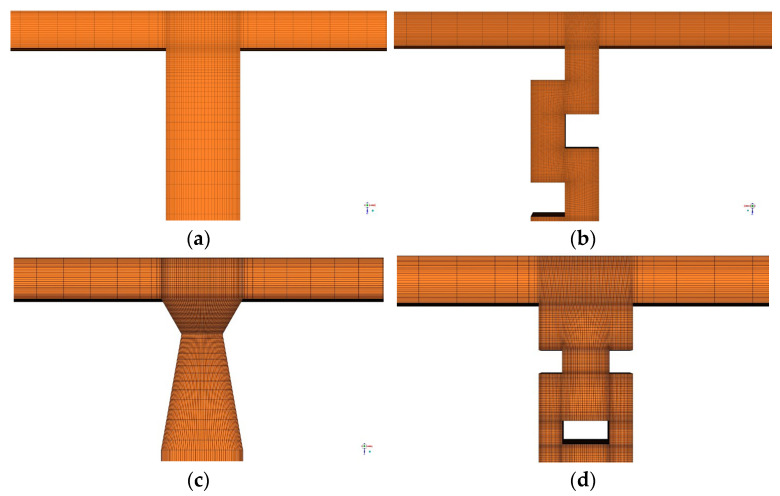
Schematic diagrams of the model mesh; (**a**) TMR; (**b**) B-TMR; (**c**) O-TMR; (**d**) V60-TMR.

Structured meshes were generated for all microreactors using ICEM. Figure 3 shows the mesh independence verification of the TMR at Re=2000. When the mesh count exceeds 180,000, the segregation index and pressure drop from the simulation remain nearly constant. Thus, a mesh model with 389,100 elements was adopted for subsequent calculations. The mesh count and quality for microreactors with different structures are listed in Table 1. Schematic diagrams of the mesh models for microreactors with different structures are shown in Figure 4.

### 2.5. Boundary Conditions and Solution Method

Due to the stability of liquid flow in microchannels, the steady-state simulation method is used in this paper. Fluid A and Fluid B enter from the two inlet channels, respectively, with velocity inlets specified at the inlets. The hydraulic diameter was calculated using *D* = 4*A/l*, where *A* is the cross-sectional area and *l* is the wetted perimeter, and the turbulence intensity was calculated using *I* = 0.16*Re*^−1/8^. The outlet was set as a pressure outlet with atmospheric pressure.

The convergence criteria were set as follows: $10^{-3}$ for the continuity and momentum equations, and $10^{-5}$ for the species transport equation. During the simulation, the concentrations of *I*_2_, *I*_3_^−^, *H*_3_*BO*_3_, and *H*^+^ were monitored. All simulations were performed using ANSYS Fluent 19.0 (ANSYS Inc., Canonsburg, PA, USA), with the discretization methods for key variables listed in Table 2.

The operating parameters and constants used in this study were listed in Table 3.

## 3. Results

### 3.1. Experimental Verification of the Microreactor

To validate the turbulence model and determine the parameters of the EDM, we conducted pressure drop and micromixing experiments in a TMR. The experimental methodology was consistent with that reported in Reference [47], and the reactant concentrations were identical to those employed in the simulations.

**Figure 5:**
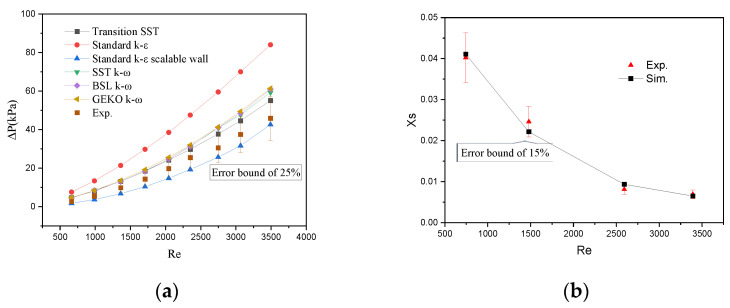
(**a**) Comparison of simulated pressure drop values (different turbulence models) with experimental data; (**b**) comparison of simulated and experimental $X_{S}$ values under different flow rates.

**Figure 6:**
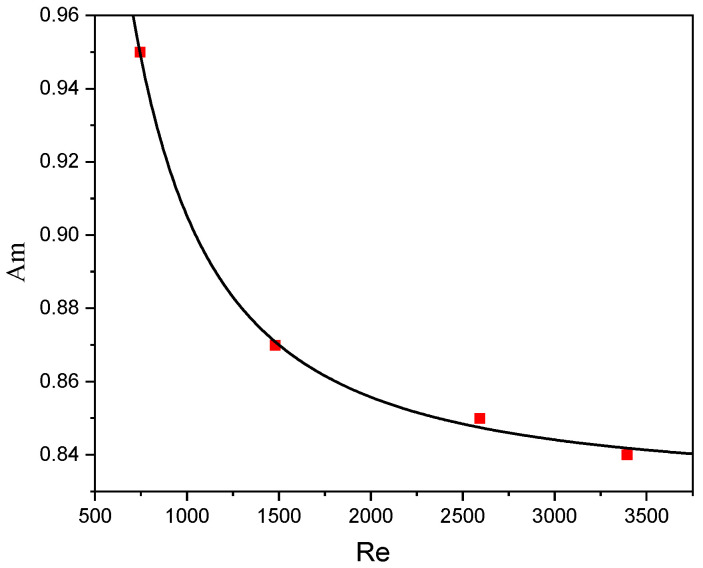
Fitting curve between *Re* and *A_m_*.

The pressure drop measured under different flow conditions was used to verify the turbulence model. Figure 5a shows the comparison of pressure drop simulation values with experimental data using different turbulence models. The standard *k-ε* model shows the largest error, while the standard *k-ε* model with an extensible wall function and the Transition SST model exhibit smaller errors. At medium-to-high Reynolds numbers (*Re* > 2000), the standard *k-ε* model with an extensible wall function shows better agreement with experimental data (a relative error ≤ 25%) and was thus adopted for subsequent simulations. Figure 5b shows that the error between the simulated and experimental *X_S_* values is within 15%. In this paper, the fitting relationship between *A_m_* and *Re* (shown in Figure 6) was used to determine *A_m_* under different flow rates, with the fitting function expressed as Equation (9) and a goodness of fit $R^{2}=0.998$.

(16) A=4571Re−1.597+0.8313

### 3.2. Flow Field Characteristics in Microreactors with Different Enhanced Structures

**Figure 7:**
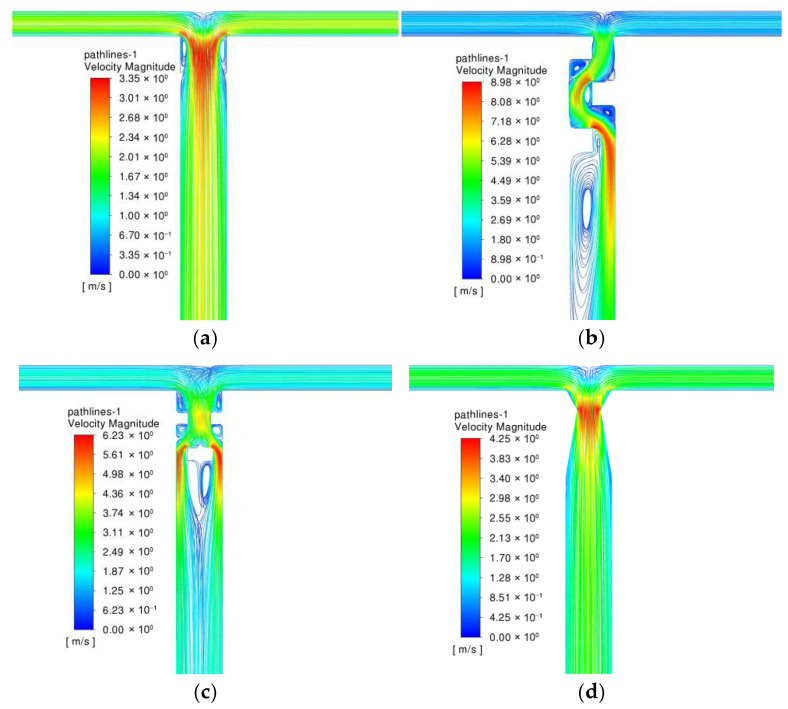
Velocity contour maps under different enhanced structures: (**a**) TMR; (**b**) B-TMR; (**c**) O-TMR; (**d**) V60-TMR.

Figure 7 shows the velocity distribution characteristics of TMRs, B-TMRs, O-TMRs, and V60-TMRs at Re=1600. In the unstructured microreactor (TMR), the two fluid streams converge and contract into the mixing channel, with a maximum velocity of 3.35 m/s. In the B-TMR, the flow velocity in the mixing channel fluctuates continuously after confluence, reaching a maximum of 8.86 m/s. In the O-TMR, the fluid undergoes sudden contraction followed by re-separation, with a maximum velocity of 6.22 m/s. In the V60-TMR, the fluid contracts and expands gradually, with a maximum velocity of 4.26 m/s (slightly higher than that of the TMR). Compared with the other three structures, the venturi’s contraction–expansion design guides fluid flow without obvious boundary layer separation, i.e., without the occurrence of obvious dead zones.

**Figure 8:**
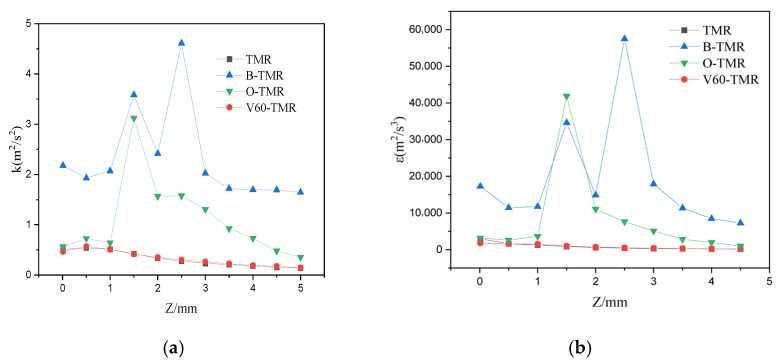
Distributions of (**a**) turbulent kinetic energy and (**b**) turbulent dissipation rate along the tube length (different enhanced structures) at *Re* = 1600.

Figure 8 shows the distribution characteristics of turbulent kinetic energy and turbulent dissipation rate along the tube length. Similarly to the velocity peaks, the baffle structure exhibits the highest peaks of turbulent kinetic energy and dissipation rate, followed by the orifice-plate structure. The V60-TMR and TMR show significantly lower values. In the first 3 mm of the mixing channel, the baffle structure exhibits two peaks (one large, one small) in turbulent kinetic energy and dissipation rate. This is because the baffle causes two major changes in flow direction and cross-sectional area, as shown by the two maximum velocity regions in the velocity contours. This indicates that the baffle and orifice-plate structures induce higher turbulence intensity due to continuous flow path perturbations in the confluence region, resulting in higher energy consumption.

**Figure 9:**
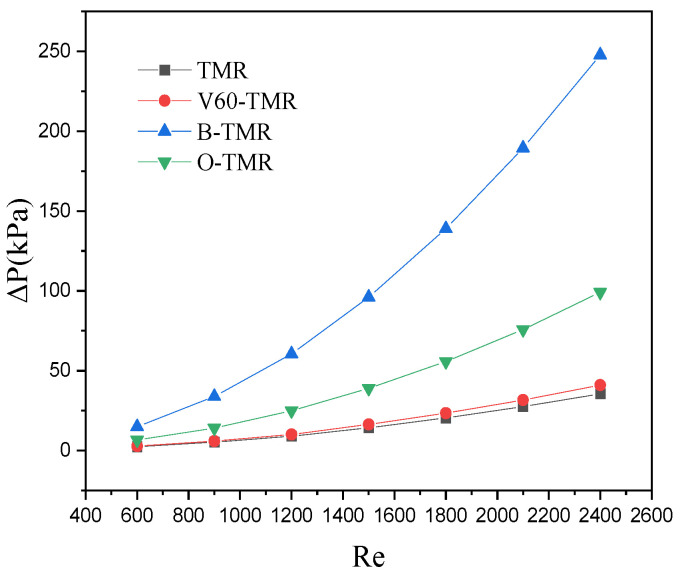
Effect of *Re* on pressure drop (different enhanced structures).

Figure 9 shows the effect of *Re* on pressure drop in four types of TMR. For all structures, pressure drop increases with *Re*: the baffle structure shows the steepest increase, while TMR and V60-TMR show gentle increases. At the same Re, the pressure drop follows the order: B-TMR > O-TMR > TMR ≈ V60-TMR.

### 3.3. Micromixing Efficiency of Microreactors with Different Enhanced Structures

**Figure 10:**
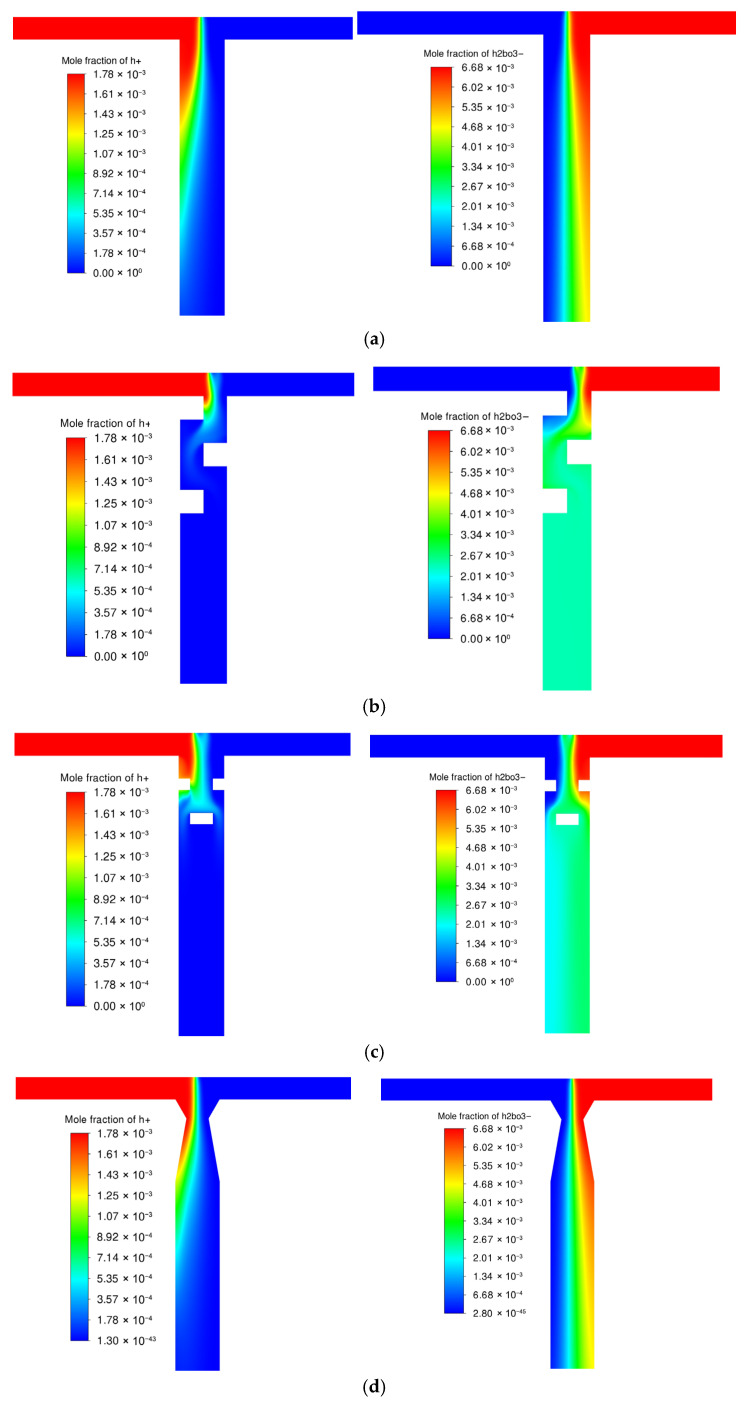
Component concentration contours under different enhanced structures at *Re* = 1600: (**a**) TMR; (**b**) B-TMR; (**c**) O-TMR; (**d**) V60-TMR; (**left**): *H*^+^; (**right**): *H*_2_*BO*_3_^−^.

Figure 10 shows the distribution characteristics of *H*^+^ and *H*_2_*BO*_3_^−^ concentrations in the four microreactors. The baffle structure induces the fastest key reactant (*H*^+^) consumption (nearly complete at 1.2 mm from the mixing point) due to high turbulence intensity. The orifice-plate structure follows, while TMR and V60-TMR show similar *H*^+^ distribution patterns (V60-TMR has a slightly faster *H*^+^ decrease). The other reactant (*H*_2_*BO*_3_^−^) achieves homogeneous mixing first in the baffle configuration, followed by the orifice plate configuration, and TMR and V60-TMR present similar *H*_2_*BO*_3_^−^ distribution patterns. As a limiting reactant, the faster the concentration of *H*^+^ ions decreases, the more *H*^+^ ions participate in Reaction R1. Correspondingly, the higher the micromixing efficiency, the lower the *X_S_*.

**Figure 11:**
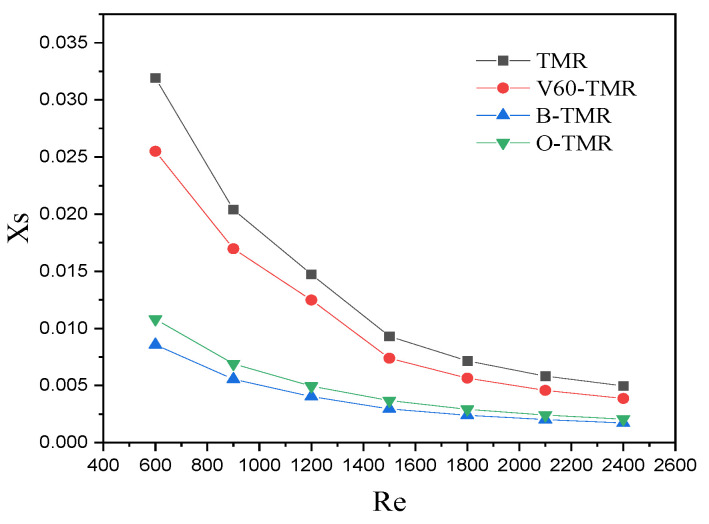
Effect of Re on $X_{S}$ (different enhanced structures).

Figure 11 shows the effect of *Re* on the *X_S_* of four TMRs. For all structures, *X_S_* decreases with increasing Re (higher turbulence intensity improves mixing). At the same Re, *X_S_* follows the order B-TMR < O-TMR < V60-TMR < TMR, indicating that the baffle structure has the best micromixing efficiency, followed by the orifice-plate structure. The baffle structure demonstrates high micromixing efficiency, which is consistent with previous studies on flow perturbation-enhanced mixing [24,26]. However, its significant pressure drop (increasing rapidly with flow rate) limits practical applicability in energy-sensitive systems. The orifice-plate structure offers moderate micromixing performance but still suffers from substantial pressure drop, attributed to sudden flow contraction and separation [34]. In contrast, the venturi structure achieves improved micromixing efficiency with minimal pressure drop increase, making it superior to the conventional TMR for industrial applications. This is because the gradual contraction–expansion design of the venturi guides fluid flow without severe boundary layer separation, reducing energy loss while enhancing turbulence [35]. Therefore, numerical simulations were conducted on microreactors with venturi structures of varying angles, and the effects of geometric angles on their hydrodynamic characteristics (e.g., pressure drop, flow field distribution) and micromixing efficiency were discussed in detail below.

Notably, compared with TMR, the venturi structure improves micromixing efficiency (lower $X_{S}$) with a minimal increase in pressure drop, and no obvious flow dead zones are observed in Figure 7d, making it more promising for practical applications.

### 3.4. Fluid Dynamics and Micromixing Efficiency of Venturi-Type Microreactors

**Figure 12:**
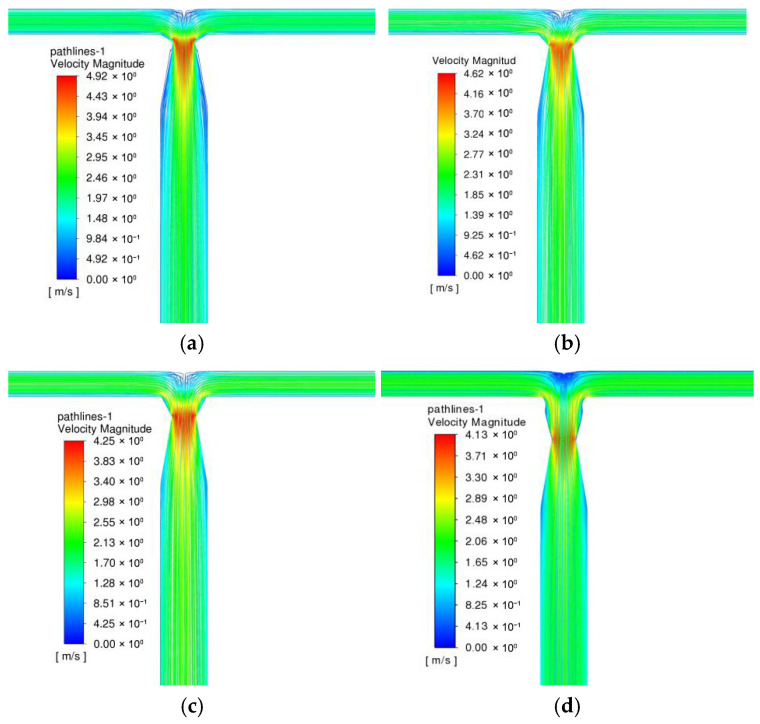
Velocity contour maps of venturi-type microreactors (different contraction angles): (**a**) V30-TMR; (**b**) V45-TMR; (**c**) V60-TMR; (**d**) V75-TMR.

Figure 12 shows the velocity distribution of venturi-type microreactors (V30-TMR, V45-TMR, V60-TMR, V75-TMR) at Re = 1600. Due to the uniform contraction port size (0.6 mm), all structures show similar velocity distribution patterns, with maximum velocities in the order: V30-TMR > V45-TMR > V60-TMR > V75-TMR. Furthermore, it can also be observed from this figure that, under operating conditions, prominent flow dead zones occur on both sides of the confluence region in the V75-TMR compared with other venturi structures.

**Figure 13:**
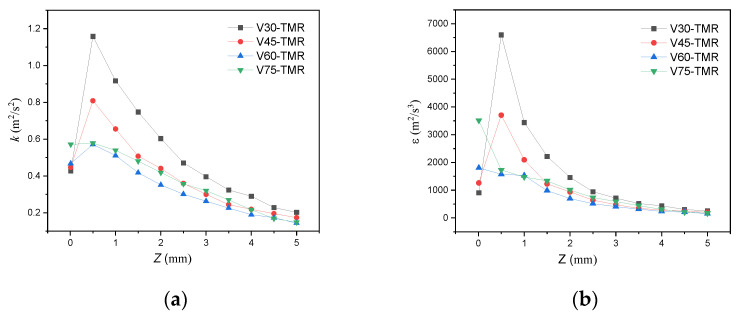
Distributions of (**a**) turbulent kinetic energy and (**b**) turbulent dissipation rate along the tube length (venturi-type microreactors) at *Re* = 1600.

**Figure 14:**
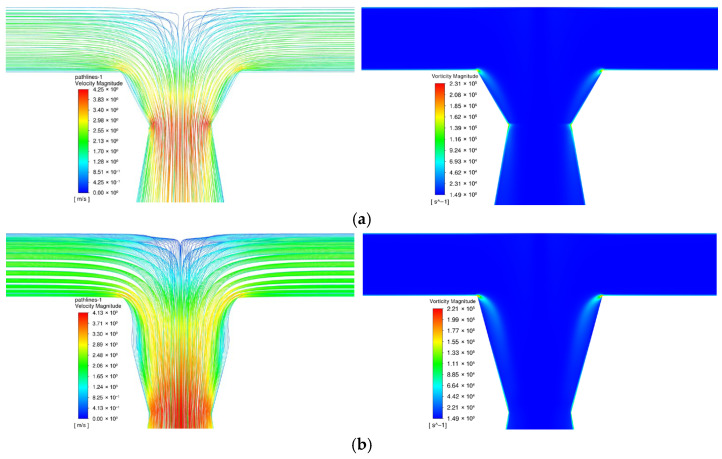
Local velocity and vorticity diagrams of venturi-type microreactor at *Re* = 1600; (**a**) V60-TMR; (**b**) V75-TMR; (**left**): velocity; (**right**): vorticity.

Figure 13 shows the distribution of turbulent kinetic energy and turbulent dissipation rate along the tube length at *Re* = 1600. Peaks of both parameters occur within the first 2 mm of the mixing channel (highest turbulence intensity after fluid collision) and gradually stabilize along the tube length. The turbulent kinetic energy peaks follow the order: V30-TMR > V45-TMR > V75-TMR > V60-TMR, while the dissipation rate peaks follow: V30-TMR > V45-TMR > V75-TMR > V60-TMR. Figure 14 presents the velocity and vorticity contours of the mixing zones in the V60-TMR and V75-TMR. Compared with venturi microreactors with a 60° contraction angles, the more gradual 75° contraction angle results in an increased length of the contraction section in the reactor and intensifies boundary layer separation on both sides of the flow channel, consequently leading to a higher maximum turbulent kinetic energy and energy dissipation rate in this region than that of the V60-TMR.

**Figure 15:**
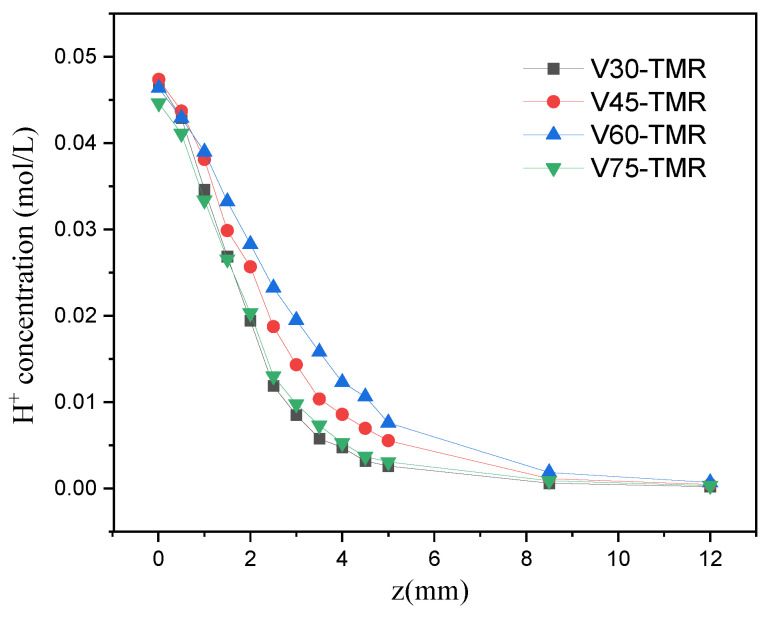
Variation in *H*^+^ concentration along the tube length (venturi-type microreactors) at *Re* = 1600.

Figure 15 shows the variation in *H*^+^ concentration along the tube length in four venturi-type microreactors with different contraction angles at *Re* = 1600. As can be observed from the figure, *H*^+^ is full consumed at the 12 mm position of the mixing channel, indicating that the reaction process is almost completed here. Furthermore, the smaller the contraction angle, the higher the turbulence intensity after fluid confluence; enhanced mixing thereby accelerates *H*^+^ consumption, resulting in a more pronounced decreasing trend of its concentration.

**Figure 16:**
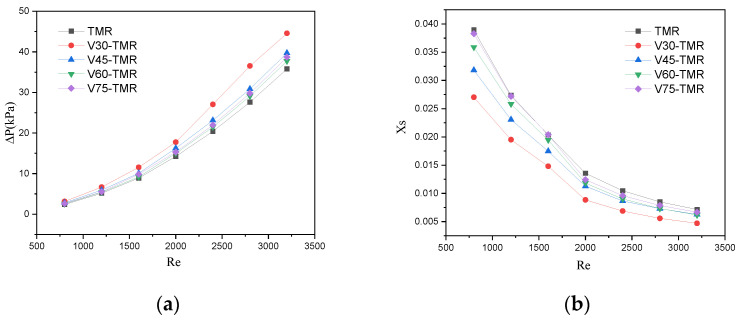
Effect of *Re* on (**a**) pressure drop and (**b**) $X_{S}$ (venturi-type microreactors and TMR).

Figure 16 shows the effect of Re on pressure drop and $X_{S}$ for venturi-type microreactors and TMR. It can be seen from Figure 16a that the pressure drop increases with the rise in *Re*. At low *Re* conditions, the pressure drops of the several microreactors show little difference. As *Re* increases, the pressure drop of V30-TMRs is significantly higher than that of the other microreactors, and the higher the *Re*, the greater the magnitude of this increase. In particular, at higher *Re*, the pressure drop of V75-TMRs is slightly higher than that of V60-TMR. The *X_S_* decreases with the increase in *Re* in Figure 16b. In the lower *Re* range, the *X_S_* follows the order: V30-TMR < V45-TMR < V60-TMR < V75-TMR. As *Re* increases, the *X_S_* of V75-TMRs decreases more rapidly and gradually approaches that of V60-TMR. In general, smaller contraction angles improve micromixing efficiency but increase pressure drop. However, the turbulence intensity and micromixing efficiency of V75-TMRs with a gentler contraction angle are more sensitive to changes in *Re*. Under the operating conditions used in this study, the V45-TMRs achieve a balance between energy consumption and mixing efficiency, thus qualifying as the preferable options among venturi configurations.

### 3.5. Micromixing Time

To better carry out a comparative analysis of the micromixing performance among various types of reactors, the agglomeration model was used to estimate the micromixing time (*t_m_*) of microreactors with different structures in this study. The model equations are as follows:

(17) $$\frac{dc_{i}}{dt}=(c_{j,A0}-c_{j})\frac{1}{g}\frac{dg}{dt}+r_{j}$$

(18) $$g(t)=exp(t/t_{m})$$

where $c_{j}$ is the concentration of component j in the mixed stream; $c_{j,A0}$ is the initial concentration of component j before mixing; $r_{j}$ is the generation rate of component j; and g is the growth rate.

**Figure 17:**
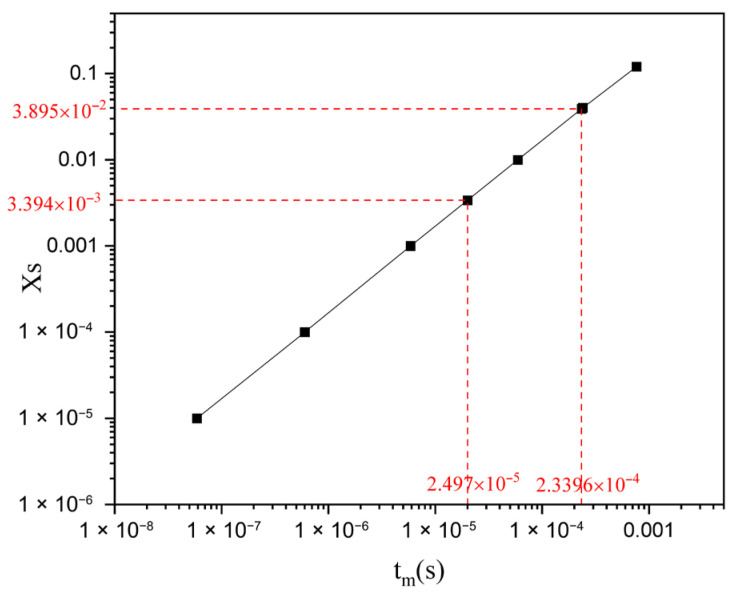
Relationship between $X_{S}$ and $t_{m}$.

**Figure 18:**
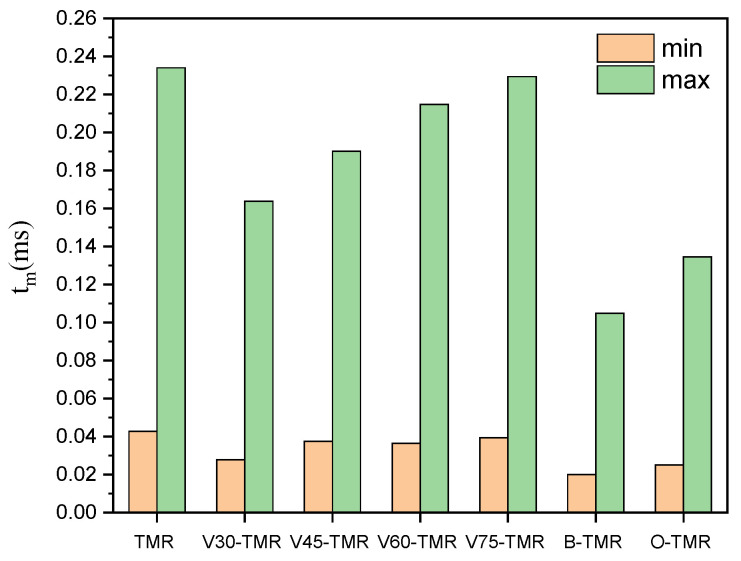
Comparison of $t_{m}$ for microreactors with different structures.

Figure 17 shows the relationship between *X_S_* and *t_m_*. The *t_m_* values of all structures range from 0.025 ms to 0.234 ms. As shown in Figure 18, the B-TMR exhibits the shortest *t_m_* among all structures, with a minimum *t_m_* approximately 53% lower than that of TMRs under the same conditions. Among venturi-type microreactors, the V30-TMR has the shortest *t_m_*, with a minimum *t_m_* approximately 35% lower than that of the TMR under the same conditions. These results indicate that structural modifications and contraction angle adjustments reduce *t_m_*.

As shown in Table 4, the $t_{m}$ calculated in this study are compared with those obtained from other milli/microreactors [48,49]. Factors affecting the *t_m_* include the experimental system, intensification measures of the milli/microreactor, operating conditions, and so on. As can be seen from the table, these *t_m_* are of a similar order of magnitude to those calculated by Gu [50] because both work systems were operated at relatively high *Re*. The key influencing factors of the $t_{m}$ in microreactors can be categorized into three types: reactor structural parameters, operational parameters, and physical property parameters of the system. In practical applications, the balance between micromixing performance and energy consumption is achieved through structural optimization and operational regulation, with the goal of obtaining a higher micromixing rate at a lower energy cost.

## 4. Conclusions

The structural design of microreactors is one of the important factors for regulating flow field characteristics and enhancing micromixing efficiency. In this study, CFD models were established to simulate the fluid flow and mixing-reaction processes in TMRs with different enhanced structures. The effects of microreactor structures on their hydrodynamic characteristics (e.g., pressure drop, flow field distribution) and micromixing efficiency were investigated under different *Re*. The main results are as follows:

The standard *k-ε* model with an extensible wall function shows the best agreement with experimental pressure drop data of the TMR, with errors within 25%. A fitting function for the *A_m_* is obtained in the *Re* range of 600–3400, and the errors between simulated and experimental *X_S_* values are within 15%.

The baffle structure generates the highest turbulence intensity and the optimal micromixing efficiency, followed by the orifice-plate structure. However, both structures suffer from significant pressure drops, which rise sharply with increasing flow rate. The venturi structure improves micromixing efficiency which minimizes the increase in pressure drop, demonstrating superior application prospects compared to the conventional TMR. Among venturi structures, the V30-TMR achieves the highest pressure drop and micromixing efficiency; the V75-TMR has more distinct flow dead zones than the V60-TMR; V45-TMRs strike a balance between energy consumption and micromixing efficiency under the operation conditions in this work.

The *t_m_* of all structures ranges from 0.025 to 0.234 ms under operational conditions. Structural optimization can shorten the *t_m_*, with the baffle structure achieving the most significant reduction (≈53% compared to TMRs). The results can provide a theoretical basis and technical guidance for optimizing the structural design of microreactors in fast reaction systems.

## Figures and Tables

**Table 1:** Mesh count and quality of each microchannel reactor.

Microreactor Type	Mesh Count	Minimum Mesh Quality
TMR	389,143	1
B-TMR	508,369	0.98
O-TMR	524,830	0.97
V30-TMR	453,816	0.87
V45-TMR	502,820	0.71
V60-TMR	410,254	0.81
V75-TMR	451,000	0.95

**Table 2:** Discretization methods for key variables.

Variable	Discretization Method
Momentum Equation	QUICK
Turbulent Kinetic Energy	Third-Order MUSCL
Turbulent Dissipation Rate	Third-Order MUSCL
Pressure–Velocity Coupling	Coupled
Pressure	PRESTO

**Table 3:** Operating parameters and constants in this study.

Parameter/Constant	Symbol	Value	Unit
Inlet flow rate	*V*_in_	16.25–92	mL/min
Reynolds number of the mixing channel	*Re*	600–3400	−
Volume flow ratio	*R*	1:1	−
Initial concentration of *H^+^*	*c*_H+,0_	0.1	mol/L
Initial concentration of *H_2_BO_3_^−^*	*c*_H2BO3−,0_	0.3636	mol/L
Initial concentration of *I^−^*	*c*_I−,0_	0.01167	mol/L
Initial concentration of *IO_3_^−^*	*c*_IO3−,0_	0.00233	mol/L
Molecular diffusion coefficient	*D*	2 × 10^−9^	m^2^/s
Density	*ρ*	997	kg/m^3^
Dynamic viscosity	*μ*	0.89	mPa·s

**Table 4:** Comparison of micromixing characteristic time.

Literature Sources	Reactor	Micromixing Intensification Method	Operational Condition	*t_m_*
This paper	T-type microreactor	Baffles, orifice-plate, venturi configuration	*Re* = 600–3400	0.025–0.234 ms
Rahimi [48]	T-type microreactor	Ultrasound wave	*Re* = 71–638	0.65–9 ms
Gu [50]	Micro-impinging stream reactor	Micro-impinging	*Rej* = 395–3161	0.02–2.1 ms
Yang [49]	Corning AFR	Heart-shaped configuration	*Q* = 1–9 mL/min	4–17 ms

## Data Availability

The data presented in this study are available on request.

## Abbreviations

The following abbreviations are used in this manuscript:

CFD	Computational Fluid Dynamics
TMR	T-Shaped Microreactor
B-TMR	Baffle-Type T-Shaped Microreactor
O-TMR	Orifice-Plate-Type T-Shaped Microreactor
V30-TMR	Venturi-Type T-Shaped Microreactor (30° Contraction Angle)
V45-TMR	Venturi-Type T-Shaped Microreactor (45° Contraction Angle)
V60-TMR	Venturi-Type T-Shaped Microreactor (60° Contraction Angle)
V75-TMR	Venturi-Type T-Shaped Microreactor (75° Contraction Angle)
PDF	Probability Density Function
EDM	Eddy Dissipation Model
*A_m_*	Empirical Constant in EDM
*X_S_*	Segregation Index
*t_m_*	Micromixing Time
*R_e_*	Reynolds Number
